# Anti-SARS-COV-2 specific immunity in HIV immunological non-responders after mRNA-based COVID-19 vaccination

**DOI:** 10.3389/fimmu.2022.994173

**Published:** 2022-08-26

**Authors:** Marta Sisteré-Oró, Naina Andrade, Diana D.J. Wortmann, Juan Du, Natalia Garcia-Giralt, María González-Cao, Robert Güerri-Fernández, Andreas Meyerhans

**Affiliations:** ^1^ Infection Biology Laboratory, Department of Medicine and Life Sciences, Universitat Pompeu Fabra (UPF), Barcelona, Spain; ^2^ Infectious Diseases Unit, Hospital del Mar, Institute of Medical Research (IMIM), Barcelona, Spain; ^3^ Instituto Oncologico Dr Rosell, Hospital Quiron-Dexeus Barcelona, Barcelona, Spain; ^4^ Department of Medicine and Life Sciences (MELIS), Universitat Pompeu Fabra (UPF), Barcelona, Spain; ^5^ Centro de Investigación Biomédica en Red Enfermedades infecciosas, CIBERINFEC Instituto de Salud Carlos III, Madrid, Spain; ^6^ ICREA, Catalan Institution for Research and Advanced Studies, Barcelona, Spain

**Keywords:** COVID-19 vaccination, SARS-CoV-2, HIV-1, immunological non-responder (INR), immunosuppression

## Abstract

Individuals infected with the human immunodeficiency virus type 1 (HIV-1) belong to the group of people most vulnerable to SARS-CoV-2 infections and the associated disease COVID-19. Here we describe SARS-CoV-2-specific antibody and cellular immune responses in a small cohort of immunological non-responder HIV-1 patients (HIV-INRs) after receiving the COVID-19 mRNA-based BioNTech/Pfizer vaccine. Compared to the control group of vaccinated healthy individuals that all developed a virus-specific immune response, 5 of 10 vaccinated HIV-1 patients showed insufficient immune responses. The lack of response was not directly correlated with patients CD4 cell counts. Three of the five non-responders that agreed to receive a booster vaccination subsequently generated a virus-specific response. Thus, even HIV-INRs can be efficiently vaccinated against COVID-19 but may require a follow-up by virus-specific immune monitoring to guarantee clinical vaccine benefits.

## Introduction

Highly efficient vaccines against the severe acute respiratory syndrome coronavirus 2 (SARS-CoV-2) pandemic have been developed with unprecedented speed. Indeed, it took merely a single year from virus sequence availability to emergency approval of the mRNA-based vaccines from BioNTech/Pfizer and Moderna (1, 2). Both vaccines induce virus spike (S) protein-specific antibodies and cellular immunity, and protect from SARS-CoV-2-induced COVID-19 disease with an efficacy of roughly 95% (1–3). However, such a vaccine efficacy is not reached in all individuals. There are groups of immune-compromised individuals that have a significantly higher mortality upon SARS-CoV-2 infection than non-compromised individuals, and that may respond less well to respective vaccines. Among these are cancer patients, transplant recipients and persons infected with human immunodeficiency viruses (4–8). Due to their immunocompromised state, these patient groups were underrepresented in the initial phase III vaccine efficacy trials (9, 10) and they deserve special attention when it comes to the evaluation of their vaccine responses (8, 11, 12).

People living with HIV-1 (PLWH) are among the group of individuals considered most vulnerable toward SARS-CoV-2 infection and its pathogenic consequences (8). They have recently been included in immunogenicity studies of mRNA-based COVID-19 vaccines (7, 10, 13–21). Respective results are summarized in **Table 1**. The majority of the PLWH in these studies were on antiretroviral therapy (ART) and had CD4 T lymphocyte counts higher than 350 per microliter blood. They usually mounted robust immune responses comparable with those of healthy controls (9, 14–16). However, some studies noted reduced vaccine responses in PLWH that had low CD4 counts (13, 19–21). Since CD4 T lymphocytes provide important help for the generation of humoral and cellular adaptive immune responses, we evaluated the response to COVID-19 vaccination in a small cohort of immunological non-responder HIV-1 patients (HIV-INRs) that are expected to be particularly susceptible and vulnerable toward SARS-CoV-2 infection. HIV-INRs represent individuals that maintain low CD4+ T cell counts despite successful HIV suppression under ART (22, 23). We report here the vaccination outcomes in a small cohort of these patients and relate them to COVID-19 vaccine studies of PLWH that have been published until April 2022 (**Table 1**) (7, 10, 13–21).

**Table 1:** Summary of HIV-related baseline characteristics and SARS-COV-2 immune responses after COVID-19 mRNA-based vaccination trials in PLWH on diverse ARTs.

Author Reference / Country	Patients [n]		Median age [years](range)		Patient received vaccine [n]		Patients on ART [n]	Antiretroviral drugs regimen	Baseline characteristics of PLWH						Co-morbidities [n]	Cut off [Unit]		Anti-S IgG titres after vaccination		Anti-RBD IgG titres after vaccination						nAbs response [n]/ total [n]		IFN-γ (SFU/million PBMCs) [median]	
	PLWH	Controls	PLWH	Controls	BNT162b2	mRNA-1273			HIV viral load (copies/mL) [n]		CD4+ T cell count (cells/μL) [n]							PLWH	Controls	CD4+ T cell count (cells/*μ*L)				All PLWH	Controls	PLWH	Controls	PLWH	Controls
	Detectable	Undetectable	<200	200-350					350-499	>500	Anti-S IgG	Anti-RBD IgG	<200	200-350		350-499	>500												
Woldemeskel et al. [14] / U.S.A	12	17	52 (25; 59)	41 (24-59)	29	0	12	Integrase Inhibitor, NNRTI, Protease Inhibitor, NRTI	3	9	0	0	0	12	n.r.	n.d.	n.d.	8.84	9.49	n.d.	n.d.	n.d.	n.d.	n.d.	n.d.	12/12	0	230	190
Levy et al. [17] / Israel	143	261	49,8 ± 11,6 (mean ± SD)	55,8 ± 14.3 (mean ± SD)	404	0	143	Integrase inhibitor- based therapy (94.4%)	7	136	3	0	0	140	16 (*)	n.d.	n.d.	n.d.	n.d.	n.d.	n.d.	n.d.	n.d.	GMT (95% CI): 5.2 (4.8-5.5)	GMT (95% CI): 6 .1 (5.8-6.4)	131/135	197/201	n.d.	n.d.
Noe et al. [13] / Germany	665	231	53 (43; 59)	n.a.	582	8	n.r	n.r.	43	622	14	651			n.r.	IgG> 34 BAU/mL	n.d.	1400 (IQR 664; 2130)	n.d.	n.d.	n.d.	n.d.	n.d.	n.d.	n.d.	n.d.	n.d.	n.d.	n.r.
Tuan et al. [16] / U.S.A	39	0	>55	n.a.	39	0	38	Integrase Inhibitor, NNRTI, Protease Inhibitor	7	32	n.r.	n.r.	n.r.	28	46 (**)	n.d.	n.d.	n.r. ; 38/39 showed positive IgG response	n.d.	n.d.	n.d.	n.d.	n.d.	n.d.	n.d.	n.d.	n.d.	n.d.	n.d.
Nault et al. [21] / Canada	106 (***)	20	43 (21; 65)	47 (21;59)	Controls: 20	PLWH: 106	106	n.r.	n.r.	n.r.	6	18		82	n.r.	n.d.	2.56 RLU	n.d.	n.d.	Mean 4.75 RLU	Mean 50.71 RLU			n.r.	n.r.	100/106	19/20	n.d.	n.d.
Ruddy et al. [20] / U.S.A	14	0	62 (56; 70)	n.a.	5	9	14	n.r.	1	13	2	1	3	8	n.r.	n.d.	IgG > 0.8 U/mL	n.d.	n.d.	n.d.	n.d.	n.d.	n.d.	>250 U/mL (all patients except for one); >239 U/mL one patient with CD4+ T cell count <200	n.d.	n.d.	n.d.	n.d.	n.d.
Jedicke et al. [7]/ Germany	52 (****)	41	60,2 (32-85) [mean (range)]	44 (23-61) [mean (range)]	93	0	52	n.r.	1	51	n.r.	n.r.	n.r.	52	n.r.	n.d.	n.d.	246.2 RU/mL (IQR 218.7)	502.5 RU/mL (IQR 118.8)	n.d.	n.d.	n.d.	n.d.	n.d.	n.d.	48/52	n.d.	n.d.	n.d.
Lombardi et al. [18]/ Italy	71	10	47 ± 8 (mean ± SD)	58 ± 8 (mean ± SD)	0	81	71	Integrase Inhibitor, NNRTI, Protease Inhibitor	5	66	0	6	7	58	7 (*****)	n.d.	n.d.	2437 U/mL IQR (1485-4526) (§)	1077 U/mL IQR(702-7551)	n.d.	n.d.	n.d.	n.d.	n.d.	n.d.	71/71	10/10	n.d.	n.d.
Milano et al. [15]/ Italy	697 (******)	0	53 (19-79)	n.a.	697	0	696	n.r.	65	632	14	683			345 (******)	n.d.	IgG > 50 AU/mL	n.d.	n.d.	n.r.	n.r.	n.r.	n.r.	7582 (44.7->200.000); (99.8%)	n.d.	n.d.	n.d.	n.d.	n.d.
Oyaert et al. [10]/ Italy	**T2**: 27; **T3:** 25 (*******)	51	47 (30-66) [mean (range)]	37 (17-63)	**T2:** 27; **T3:** 25	0	n.r.	n.r.	n.r.	n.r.	n.a.	25	n.a.	n.a.	n.r.	IgG > 33.8 BAU/mL	n.d.	**T2**: Median (BAU/mL, min-max) 3140 (200-22400); **T3**: Median (BAU/mL, min-max) 788 (75.4-8860)	**T2**: Median (BAU/mL, min-max) 3455 (674-25400); **T3**: Median (BAU/mL, min-max) 1320 (104-8330)	n.r.	n.r.	n.r.	n.r.	n.d.	n.d.	n.d.	n.d.	**T3**: 68% patients responded (17/25)	**T3**: 88.2% patients responded (45/51)

Abbreviations: PLWH, people living with Human Immunodeficiency Virus (HIV); ART, antiretroviral therapy; n, numbers; IQR, interquartile range; SD: standard deviation; GMT, geometric mean titres; IC, confidence interval; IU: international units; RLU, relative luminescence units; BAU, binding antibody unit; RLU, relative luminiscence units; SFU, spot forming units; PBMC, peripheral blood mononuclear cell; NNRTI, nonnucleotide/nonnucleoside reverse transcriptase inhibitor, nAbs, neutralizing antibodies; anti-RBD: anti-receptor binding domain protein; anti-S, anti-Spike; IFN-γ, interferon-γ; Ag, antigen; n.r, non-reported; n.d, not done; n.a., not applicable

(*) Co-morbidities included hypertension, diabetes mellitus, dyslipidaemia, ischaemic heart disease, chronic obstructive pulmonary disease, kidney disease and liver disease.

(**) Co-morbidities included cancer or other immunosuppressive conditions, diabetes, cardiovascular disease, lung disease, advanced liver disease, chronic kidney disease.

(***) While 121 participants were recruited, only 106 were analized due to that 11 had anti-COVID19 antibodies at baseline.

(*****) Co-morbidities included dyslipidemia, hypertension, Hepatitis B, Hepatitis C, diabetes, renal and cardiovascular diseases and history of neoplasms.

(****) While 88 patients were recruited in the study, only 52 patients received the two doses of the vaccine.

(******) 697 patients were analyzed at prime time-point; 577 analyzed at the boost time-point and 491 at the post-second boost time-point. Co-morbidities included dyslipidaemia, hypertension, diabetes, cardiac and/or vascular disease and chronic obstructive pulmonary disease (COBD).

(*) Co-morbidities included hypertension, diabetes mellitus, dyslipidaemia, ischaemic heart disease, chronic obstructive pulmonary disease, kidney disease and liver disease.

(**) Co-morbidities included cancer or other immunosuppressive conditions, diabetes, cardiovascular disease, lung disease, advanced liver disease, chronic kidney disease.

(*******) Analysis of the response was analyzed after 10 to 14 days (T2) and 3 months (T3) after administration of the second vaccine dose.

(*******) Analysis of the response was analyzed after 10 to 14 days (T2) and 3 months (T3) after administration of the second vaccine dose.

## Materials and methods

### Sample collection and participant characteristics

This is an observational study that collected data and blood samples from HIV-INRs that were vaccinated with the mRNA-based COVID-19 vaccine BNT162b2 from BioNTech/Pfizer following the standard schedule (prime at day 0 and boost after 21 days). The study included a control cohort of healthy individuals vaccinated alike. The primary endpoint was to describe the specific IgG serum antibody response, the virus neutralizing capacity of these antibodies, and the T cell response. The study analyzed blood samples pre-vaccination, post-vaccination, and 3 weeks (w) after an additional boost in 3 previous non-responders. Immunological assays were performed as previously reported (24). The protocol of the study was approved by the institutional review boards of the “Hospital del Mar” and “Grupo Hospitalario Quirón Salud-Catalunya”, respectively. Written informed consent was obtained from each study participant.

Blood samples from 10 HIV-INR patients and 10 healthy control individuals were collected, and sera and peripheral blood mononuclear cells (PBMCs) were isolated by standard procedures. Sampling time-points were prior to BNT162b2 vaccination and 3 weeks (w) after. Five patients (P4; P5; P6; P8; P9) did not generate responses above the threshold. Of these, P4, P5 and P9 gave consent for an additional vaccination boost with BNT162b2. From these, blood was again sampled 3 w after the 3^rd^ BNT162b2 vaccine dose. Patients P6 and P8 did not consent to receive the 3^rd^ vaccination.

Participant characteristics were as follows: HIV-INR: the median age of the 10 patients was 49 years (IQR, 30 – 71); 7 (70%) were male and none had a pre-vaccination history of COVID-19. During the study, all patients were on ART for ≥6 months and 8 (80%) had an undetectable HIV viral load. Eight (80%) had CD4 counts <200 cells/mm^3^, whereas 2 (20%) had CD4 counts of 200-349 cells/mm^3^. The overall characteristics are further specified in **Table 2**.

**Table 2:** HIV-INR patient characteristics.

Patient	Gender	Age	Current Coinfection	History of Oportunistic Infection	Age of HIV Diagnosis	Year starging ART	Time undetectable (years)	Current Viral load (copies/ml)	Current CD4+T-cell count	Nadir CD4+T-cell count	CDC Stage
Patient 1	Male	68	No	Yes	1993	1998	20	ILD	186	83	C3
Patient 2	Female	30	No	No	2017	2017	4	363	171	75	B3
Patient 3	Male	35	No	Yes	2016	2016	4	ILD	87	30	C3
Patient 4	Female	55	No	Yes	1995	2015	5	ILD	79	60	C3
Patient 5	Female	71	No	Yes	1992	1998	15	ILD	293	67	C3
Patient 6	Male	41	Yes (Hepatitis B)	No	2008	2017	4	ILD	318	180	B3
Patient 7	Male	49	No	Yes	2010	2010	11	ILD	153	33	C3
Patient 8	Male	42	No	No	2006	2015	0	211	32	12	B3
Patient 9	Male	55	No	No	2016	2016	5	ILD	77	77	B3
Patient 10	Male	52	Yes (Hepatitis B)	Yes	2008	2008	1	ILD	165	54	B3

ILD, inferior to limit of detection.

Healthy control individuals: the median age of the 10 individuals was 47 years (IQR, 26 – 72); 4 (40%) were male and none had a pre-vaccination history of COVID-19.

### Quantification of SARS-CoV-2 Spike-specific IgG antibody responses and RBD neutralization capacity of patient sera

To quantify IgG antibodies against the full-length Spike protein of SARS-COV-2, 96-well high-binding plates (2240096, BioRad) were coated with 2 μg/mL (Sino Biologicals, 40589-V08B1). After a washing step, plates were blocked with 3% BSA (A4503, Sigma-Aldrich) in 1x PBS for 1 h at room temperature (RT). Next, three-fold dilutions of patients’ sera were added in duplicates and incubated for 1 h at RT. Subsequently, washed plates were dispensed with IgG-HRP antibody (A18811, Life technologies) and left incubating for 1 h at RT. Lastly, the 3,3’,5,5’-Tetramethylbenzidine **(**TMB) substrate solution was added and stopped with 1 N H_2_SO_4_. Optical densities (OD) values of the plates were measured at 450 nm wavelength using a microplate reader (iMark Microplate Reader, Bio-Rad). For each dilution of the serum sample duplicates, the mean value absorbance was calculated. The endpoint-titer of the IgG Spike-specific binding antibody titers were determined as the reciprocal of the last serum dilution which provided 3 times the mean OD of the negative control (wells with media only). Endpoint-titers above a 1:2700 dilution were considered as positive.

To quantify neutralizing antibodies against the Receptor Binding Domain (RBD) of the Spike protein of SARS-COV-2, a SARS-CoV-2 NeutraLISA assay (EI 2606-9601-4, Euroimmune) was run with the collected patients´ sera according to the manufacturer’s instructions. Samples were always analyzed in duplicates and absorption values measured at 450 nm by means of a microplate reader (iMark Microplate Reader, BIO-RAD). Mean of the duplicates was calculated and final percentages of inhibition (% IH) calculated as follows: 100% - [(extinction sample x 100%)/average extinction blank]. Values were considered positive when % IH reached ≥35%, doubtful when % IH were between ≥20 to <35 and negative when % IH<20.

### Quantification of Spike-specific T cells from PBMCs

Spike-specific T cells were detected and quantified by using IFN-γ ELISpot kits (3420-2H, Mabtech) according to the manufacturer’s instructions. In brief, 2,5 x 10^5^ PBMCs were seeded per well and *ex vivo* stimulated for 16-24h with Spike-overlapping peptides (JPT-PM-WCPV-S, SinoBiologicals, 2 µg/mL). Positive controls consisted of PBMCs incubated with PMA (P8139, Sigma, 15 ng/mL) plus ionomycin (I0634, Sigma, 250 ng/mL) and PBMCs incubated with CEF peptide pools (JPT-PM-CEF-S1, SinoBiologicals, 2 µg/mL). All samples were run in duplicates. A dissection microscope was used (Leica GZ6) for counting the number of spots obtained. To quantify antigen-specific responses, mean spots of the RPMI control wells were subtracted from the positive wells, and the results were expressed as spot-forming units (SFU) per 10^6^ PBMCs. Specific T cell responses were considered as positive if numbers were over 18 IFN-γ-secreting cells per million PBMCs.

### Statistical analysis

Statistical analyses of all data were performed using GraphPad PRISM v9. To determine *P* value and to compare the means, the Mann-Whitney-U and Kruskal Wallis test were conducted. *P* values < 0.05 were considered significant.

## Results

Twenty individuals participated in this study. Ten of these designated as P1 to P10 were HIV-INRs with ART-controlled HIV-1 loads and CD4 T lymphocyte counts below 350 per µL blood (**Table 2**). The other 10 individuals were healthy controls (HC). No previous infection by SARS-CoV-2 was recorded at inclusion in the study. All the participants received two doses of the BNT162b2 vaccine from BioNTech/Pfizer (prime at day 0 and boost after 21 days).

All HIV-INRs were out-patients of the Infectiology Unit of the Hospital del Mar in Barcelona, Spain. They were under steady antirretroviral treatment. There were no changes in HIV infection control (no changes in CD4 T-cell counts or viral loads) related to vaccination. No major side effects due to vaccination were identified in any of these inidividuals.

**Figure 1:**
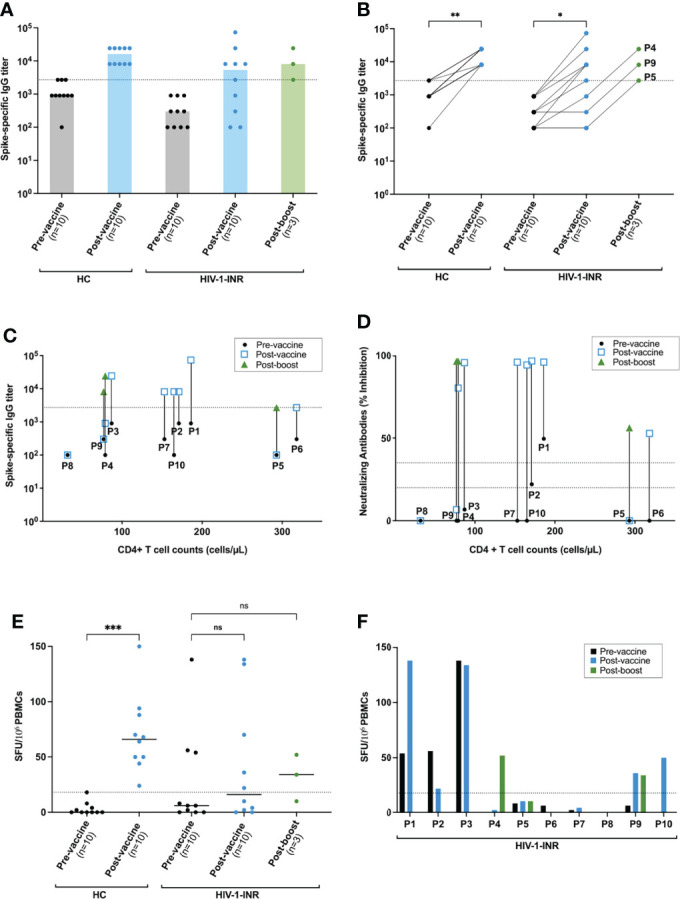
Spike-specific anti-SARS-CoV-2 antibody and cellular responses in HIV-INR and healthy donors after mRNA-based COVID vaccination. **(A, B)** IgG Spike-specific titers determined by ELISA for both of the 2 cohorts: healthy controls (HC) and HIV-INR at the 3 defined time-points: pre-vaccination (pre-vaccine), post-vaccination (post-vaccine) and, for the HIV-INR non-responder patients, 3 w post boost vaccination (post-boost). IgG Spike-specific titers of the HIV-INR **(C)** and individual inhibition percentages (% IH) against RBD determined by NeutraLISA **(D)** of all the HIV-INR patients taking into account their individual CD4+ T cell counts at the 3 different time-points. **(E)** IFN-γ ELISpot values (SFU per 10^6^ PBMCs) for all healthy individuals (HC) and HIV-INR patients at the 3 different time-points after *in vitro* stimulation with overlapping Spike peptides of SARS-CoV-2. **(F)** IFN-γ ELISpot values for individual patients. Differences between the groups were calculated using Mann–Whitney test or Kruskal-Wallis test for comparison of two groups. Non-significant differences were indicated as “ns”. P-values below 0.05 were considered significant and were indicated by asterisks: *p< 0.05; **p < 0.01; ***p < 0.001.

To quantify SARS-CoV-2-specific humoral immune responses after BNT162b2 vaccination, ELISAs against the Spike protein (S) of the SARS-CoV-2 Wuhan isolate were carried out before (pre-vaccine) and 3 w after vaccination (post-vaccine). Virus-specific median ELISA IgG antibody titres significantly increased after vaccination of the healthy controls (HC) (*P*<0.001) however, this was not as much significant for the vaccinated HIV-INR cohort (P<0.05) (**Figures 1A, B**). For the latter, only 5 of the 10 individuals generated specific IgGs above the threshold of positivity (**Figure 1B**). Together this supports the known strong immunogenicity of BNT162b2 and reveals the immunocompromised state of some of the HIV-INR patients.

To test whether the 5 non-responder patients P4, P5, P6, P8 and P9 would benefit from an additional vaccine boost, they were offered a 3^rd^ BNT162b2 vaccination. Three of them, P4, P5 and P9, gave consent and were vaccinated and assayed as before. Importantly, all 3 individuals responded favorably and increased their S-specific IgG antibody titres (**Figure 1B**). Thus, despite being immunocompromised, HIV-INR patients may still generate humoral immunity against novel antigens when sufficiently boosted.

To analyze whether the capacity to adequately respond to vaccination was linked to HIV-INRs´ CD4 T lymphocyte counts, the induced S-specific IgG titres were plotted as a function of patients CD4 cell counts at the time of receiving the first and subsequent vaccine shots (**Figure 1C**). No clear correlation between the vaccine response and CD4 T cell counts were observed. Three of the 5 vaccine non-responders (P4, P8 and P9) had CD4+ T cell counts below 200 cells/μL blood, the other two (P5 and P6) were close to 300 CD4+ T cells/μL (**Figure 1C**). Thus, the CD4 T cell count alone does not serve as a simple predictor of vaccine responsiveness for HIV-INRs.

To further access vaccine-induced neutralizing antibodies, SARS-CoV-2 NeutraLISA assays were performed from the sera of all study participants. The percentages of inhibition (% IH) values for the HIV-INRs at the pre-vaccine, post-vaccine and for P4, P5 and P9 also at the post-boost time points are represented in **Figure 1D**. Importantly, all healthy controls and all 5 HIV-INRs that generated S-specific IgG titres by ELISA after the standard vaccination schedule also generated strong neutralizing antibody responses with % IH values close to 100%. In contrast, the 5 post-vaccine ELISA non-responders generated none (P5, P8 and P9 with % IH values of 0, 0 and 6,62%, respectively) or reduced neutralizing antibody responses (P4 and P6 had positive % IH values of 81 and 53, respectively). Interestingly, upon vaccine boost, the neutralizing antibody response improved in all 3 vaccinees, reaching close to 100% IH values for P4 and P6, and 56% for P5. Together this demonstrates an important heterogeneity within the HIV-INR patient group with respect to humoral vaccine responses and supports additional booster vaccinations for those who have a reduced immunocompetence.

To finally access BNT162b2 vaccine-induced S-specific cellular immune responses, IFN-γ ELISpot assays with overlapping S protein-derived peptides were performed. The results are presented as IFN-γ Spot Forming Units (SFU) per 10^6^ PBMCs in **Figures 1E, F**. The standard vaccination schedule resulted in a significant increase of S-specific IFN-γ-producing T cells within the HC cohort (P< 0.001) (**Figure 1E**). SFUs before vaccination were below or at our threshold of positivity. After vaccination, SFUs were increased and spread out between 24 per 10^6^ PBMCs, which was just above our threshold of positivity, to 150 SFUs per 10^6^ PBMCs. Within the HIV-INR cohort, 3 patients (P1, P2 and P3) already had clear S-specific T cell responses before vaccination, possibly due to previous SARS-CoV-2 or other coronavirus exposures. All 3 generated high S-specific IgG titres and neutralizing antibody responses after BNT162b2 vaccination. The other 7 HIV-INRs showed no pre-vaccine T cell response. Vaccination increased specific T cells above threshold levels in just 2 of these 7 patients (P9 and P10). Boosting then increased T cells in an additional patient (P4). For patients P5, P6, P7 and P8, no Spike–specific T cells above the threshold were observed. Three of these (P5, P6 and P8) were among the antibody non-responders after the standard vaccination scheme. Boosting of P5 did not increase specific T cells. However, it resulted in a measurable but still lower than optimal neutralizing antibody response (**Figure 1D**). Thus, HIV-INR patients are quite heterogenous with respect to vaccine-specific cellular T cell responses. In some cases, low cellular responses are linked to low humoral responses.

## Discussion

Our study demonstrates a significant impairment of adequately generating SARS-CoV-2-specific immune responses after COVID-19 vaccination in about 50% of HIV-INR patients. Vaccine responsiveness was not directly linked to patients´ CD4 T cell counts. Vaccine boosters improved the specific responses. Thus, to provide optimal SARS-CoV-2-preventive health care for this vulnerable patient group, the level of vaccine-induced immune responses should be followed by diagnostic assays and booster vaccination should be offered if antibody levels are low.

To our knowledge, this is the first study that evaluated COVID-19 vaccination in HIV-INR patients. This particular group of HIV-1-infected individuals may represent around 20% of PLWH and is characterized by well-controlling HIV-1 replication after antiretroviral treatment however failing to improve CD4 T lymphocyte numbers above 350 cells per μL blood (23). This low CD4 T cell numbers represent a shortage of helper cells whose main function is to coordinate adaptive immune responses. Thus, a reduced adaptive immune response after vaccination of HIV-INRs is expected. The observed percentage of non-responders of about 50% is at the high end of that found for other immunocompromised patient groups like HIV-infected individuals in general (around 4%; see **Table 1**) or solid cancer patients (around 10%) (25, 26), and approaches that of transplant recipients (>50%) (27) and some hematological cancers (around 60%) (28).

Several COVID-19 vaccination studies with HIV-1 infected individuals have recently been carried out (7, 10, 13–21). Their main results are summarized in **Table 1**. The majority of the participants of these studies had high CD4 T cells counts. They generated efficient SARS-CoV-2-specific immune responses and did not experience important adverse effects. However, a few of the study participants had low CD4 T cell counts (13, 15, 17–21). Within this subgroup, some of them generated diminished humoral immune responses. Their responses after a vaccine boost have not been described.

A limitation of our study is the low number of HIV-INR study participants. Nonetheless, since 50% of them markedly fail to generate SARS-CoV-2-specific immune responses, this is a clear indication of the general immunocompromised state of this special patient cohort. Importantly, vaccine non-responsiveness comprised the humoral as well as the cellular arm of immunity even though there was not a complete overlap. Since neutralizing antibodies and cytotoxic T cell responses are considered to act multiplicative against virus infections (29), also unbalanced vaccine responses might put individuals at risk of severe infection outcomes. Furthermore, lack of sufficient helper T cells in time of vaccination may significantly reduce the duration of the specific memory response (30). Additional studies along these lines are highly warranted.

In conclusion, mRNA-based COVID-19 vaccines are effective in inducing immune responses in some HIV-INR patients. Due to the high percentage of vaccine non-responders, preventive health care measures like vaccine response monitoring and booster vaccinations for this special cohort against SARS-CoV-2 are indicated.

## Data availability statement

The original contributions presented in the study are included in the article/supplementary material. Further inquiries can be directed to the corresponding authors.

## Ethics statement

The studies involving human participants were reviewed and approved by “Hospital del Mar” and “Grupo Hospitalario Quirónsalud-Catalunya”. The patients/participants provided their written informed consent to participate in this study.

## Author contributions

Concept and funding acquisition: RG-F and AM. Experimental design: MS-O, RG-F, and AM. Experiment performance: MS-O, NA, DDJW, and JD. Patient recruitment and handling: NG-G, MG-C, and RG-F. Sample collection: JD and MS-O. Figures and tables: MS-O, NA, DDJW, and RG-F. Manuscript drafting: MS-O and NA with corrections from AM and RG-F. All authors contributed to the article and approved the submitted version.

## Funding

The authors are supported by Spanish Melanoma Group Grant (GEM) (III Beca GEM para Grupos Emergentes), the Spanish Ministry of Science and Innovation grant no. PID2019-106323RB-I00 AEI//10.13039/501100011033, the “Unidad de Excelencia María de Maeztu”, funded by the MCIN and the AEI (DOI: 10.13039/501100011033); Ref: CEX2018-000792-M and by a FIS Project “PI19/00019” funded by Instituto de Salud Carlos III (ISCIII) and co-funded by the European Union.

## Conflict of interest

The authors declare that the research was conducted in the absence of any commercial or financial relationships that could be construed as a potential conflict of interest.

## Publisher’s note

All claims expressed in this article are solely those of the authors and do not necessarily represent those of their affiliated organizations, or those of the publisher, the editors and the reviewers. Any product that may be evaluated in this article, or claim that may be made by its manufacturer, is not guaranteed or endorsed by the publisher.

## References

[1] Pascual-Iglesias A Canton J Ortega-Prieto AM Jimenez-Guardeño JM Regla-Nava JA. An overview of vaccines against SARS-COV-2 in the COVID-19 pandemic era. Pathogens (2021) 10(8):1030. doi: 10.3390/pathogens10081030 34451494 PMC8402174

[2] Dolgin E. The tangled history of mRNA vaccines. Nature (2021) 597(7876):318–24. doi: 10.1038/d41586-021-02483-w 34522017

[3] Tregoning JS Flight KE Higham SL Wang Z Pierce BF. Progress of the COVID-19 vaccine effort: viruses, vaccines and variants versus efficacy, effectiveness and escape. Nat Rev Immunol (2021) 21(10):626–36. doi: 10.1038/s41577-021-00592-1 PMC8351583 34373623

[4] Cavanna L Citterio C Toscani I. COVID-19 vaccines in cancer patients. seropositivity and safety. systematic review and meta-analysis. Vaccines (2021) 9(9):1048. doi: 10.3390/vaccines9091048 34579285 PMC8473083

[5] Roeker LE Knorr DA Thompson MC Nivar M Lebowitz S Peters N. COVID-19 vaccine efficacy in patients with chronic lymphocytic leukemia. Leukemia (2021) 35(9):2703–5. doi: 10.1038/s41375-021-01270-w PMC8118367 33986431

[6] Rincon-Arevalo H Choi M Stefanski A-L Halleck F Weber U Szelinski F. Impaired humoral immunity to SARS-CoV-2 BNT162b2 vaccine in kidney transplant recipients and dialysis patients. Sci Immunol (2021) 6(60):eabj1031. doi: 10.1126/sciimmunol.abj1031 34131023

[7] Jedicke N Stankov MV Cossmann A Dopfer-Jablonka A Knuth C Ahrenstorf G. Humoral immune response following prime and boost BNT162b2 vaccination in people living with HIV on antiretroviral therapy. HIV Med (2022) 23(5):558–63. doi: 10.1111/hiv.13202 PMC8652991 34725907

[8] Galmiche S Luong Nguyen LB Tartour E de Lamballerie X Wittkop L Loubet P. Immunological and clinical efficacy of COVID-19 vaccines in immunocompromised populations: a systematic review. Clin Microbiol Infect (2022) 28(2):163–77. doi: 10.1016/j.cmi.2021.09.036 PMC8595936 35020589

[9] Duly K Farraye FA Bhat S. COVID-19 vaccine use in immunocompromised patients: A commentary on evidence and recommendations. Am J Heal Pharm (2022) 79(2):63–71. doi: 10.1093/ajhp/zxab344 PMC8499782 34455440

[10] Oyaert M De Scheerder MA Van Herrewege S Laureys G Van Assche S Cambron M. Evaluation of humoral and cellular responses in SARS-CoV-2 mRNA vaccinated immunocompromised patients. Front Immunol (2022) 13:858399. doi: 10.3389/fimmu.2022.858399 35401575 PMC8988283

[11] Sun L Surya S Le AN Desai H Doucette A Gabriel P. Rates of COVID-19–related outcomes in cancer compared with noncancer patients. JNCI Cancer Spectr (2021) 5(1):pkaa120. doi: 10.1093/jncics/pkaa120 33554040 PMC7853171

[12] Gao Y Chen Y Liu M Shi S Tian J. Impacts of immunosuppression and immunodeficiency on COVID-19: A systematic review and meta-analysis. J Infect (2020) 81(2):e93–5. doi: 10.1016/j.jinf.2020.05.017 PMC7228685 32417309

[13] Noe S Ochana N Wiese C Schabaz F Von Krosigk A Heldwein S. Humoral response to SARS-CoV-2 vaccines in people living with HIV. Infection (2022) 50(3):617–23. doi: 10.1007/s15010-021-01721-7 PMC8543429 34694595

[14] Woldemeskel BA Karaba AH Garliss CC Beck EJ Wang KH Laeyendecker O. The BNT162b2 mRNA vaccine elicits robust humoral and cellular immune responses in people living with human immunodeficiency virus (HIV). Clin Infect Dis (2022) 74(7):1268–70. doi: 10.1093/cid/ciab648 PMC8406881 34293114

[15] Milano E Ricciardi A Casciaro R Pallara E De Vita E Bavaro DF. Immunogenicity and safety of the BNT162b2 COVID-19 mRNA vaccine in PLWH: A monocentric study in bari, Italy. J Med Virol (2022) 94(5):2230–6. doi: 10.1002/jmv.27629 PMC9015486 35106771

[16] Tuan JJ Zapata H Critch-Gilfillan T Ryall L Turcotte B Mutic S. Qualitative assessment of anti-SARS-CoV-2 spike protein immunogenicity (QUASI) after COVID-19 vaccination in older people living with HIV. HIV Med (2022) 23(2):178–85. doi: 10.1111/hiv.13188 PMC8652674 34632695

[17] Levy I Wieder-Finesod A Litchevsky V Biber A Indenbaum V Olmer L. Immunogenicity and safety of the BNT162b2 mRNA COVID-19 vaccine in people living with HIV-1. Clin Microbiol Infect (2021) 27(12):1851–5. doi: 10.1016/j.cmi.2021.07.031 PMC8382485 34438069

[18] Lombardi A Butta GM Donnici L Bozzi G Oggioni M Bono P. Anti-spike antibodies and neutralising antibody activity in people living with HIV vaccinated with COVID-19 mRNA-1273 vaccine: a prospective single-centre cohort study. Lancet Reg Heal - Eur (2022) 13:100287. doi: 10.1016/j.lanepe.2021.100287 PMC8694797 34961855

[19] Ruddy JA Boyarsky BJ Werbel WA Bailey JR Karaba AH Garonzik-Wang JM. Safety and antibody response to the first dose of severe acute respiratory syndrome coronavirus 2 messenger RNA vaccine in persons with HIV. AIDS (2021) 35(11):1872–4. doi: 10.1097/QAD.0000000000002945 PMC10323866 33993131

[20] Ruddy JA Boyarsky BJ Bailey JR Karaba AH Garonzik-Wang JM Segev DL. Safety and antibody response to two-dose SARS-CoV-2 messenger RNA vaccination in persons with HIV. AIDS (2021) 35(14):2399–401. doi: 10.1097/QAD.0000000000003017 34261097 PMC10323870

[21] Nault L Marchitto L Goyette G Tremblay-sher D Fortin C Martel-Laferrière V. Covid-19 vaccine immunogenicity in people living with HIV-1. Vaccine (2022) 40(26):3633–7. doi: 10.1016/j.vaccine.2022.04.090 PMC9069249 35568588

[22] Battegay M Nüesch R Hirschel B Kaufmann GR. Immunological recovery and antiretroviral therapy in HIV-1 infection. Lancet Infect Dis (2006) 6(5):280–7. doi: 10.1016/S1473-3099(06)70463-7 16631548

[23] Rb-Silva R Goios A Kelly C Teixeira P João C Horta A. Definition of immunological nonresponse to antiretroviral therapy: A systematic review. J Acquir Immune Defic Syndr (2019) 82(5):452–61. doi: 10.1097/QAI.0000000000002157 31592836

[24] Sisteré-Oró M Wortmann DDJ Andrade N Aguilar A Mayo de las Casas C Casabal FG. Anti-SARS-CoV-2 immunity in long lasting responders to cancer immunotherapy through mRNA-based COVID-19 vaccination. Front Immunol (2022) 13:908108. doi: 10.3389/fimmu.2022.908108 35911701 PMC9330498

[25] Di Noia V Pimpinelli F Renna D Barberi V Maccallini MT Gariazzo L. Immunogenicity and safety of COVID-19 vaccine BNT162b2 for patients with solid cancer: A large cohort prospective study from a single institution. Clin Cancer Res (2021) 27(24):6815–23. doi: 10.1158/1078-0432.CCR-21-2439 34583970

[26] Goshen-Lago T Waldhorn I Holland R Szwarcwort-Cohen M Reiner-Benaim A Shachor-Meyouhas Y. Serologic status and toxic effects of the SARS-CoV-2 BNT162b2 vaccine in patients undergoing treatment for cancer. JAMA Oncol (2021) 7(10):1507–13. doi: 10.1001/jamaoncol.2021.2675 PMC8267843 34236381

[27] Caillard S Thaunat O. COVID-19 vaccination in kidney transplant recipients. Nat Rev Nephrol (2021) 17(12):785–7. doi: 10.1038/s41581-021-00491-7 PMC8475856 34580488

[28] Herishanu Y Avivi I Aharon A Shefer G Levi S Bronstein Y. Efficacy of the BNT162b2 mRNA COVID-19 vaccine in patients with chronic lymphocytic leukemia. Blood (2021) 137(23):3165–73. doi: 10.1182/blood.2021011568 PMC8061088 33861303

[29] Bocharov G Grebennikov D Argilaguet J Meyerhans A. Examining the cooperativity mode of antibody and CD8+ T cell immune responses for vaccinology. Trends Immunol (2021) 42(10):852–5. doi: 10.1016/j.it.2021.08.003 34561159

[30] Ahrends T Busselaar J Severson TM Bąbała N de Vries E Bovens A. CD4+ T cell help creates memory CD8+ T cells with innate and help-independent recall capacities. Nat Commun (2019) 10(1):5531. doi: 10.1038/s41467-019-13438-1 31797935 PMC6892909

